# HQML-ICU: hybrid quantum-classical machine learning for ICU mortality prediction using MIMIC-IV electronic health records

**DOI:** 10.3389/fncom.2026.1900553

**Published:** 2026-10-09

**Authors:** Abdel-Haleem Abdel-Aty, Heba Kadry, Nadir Omer, Shaaban M. Shaaban, Huda Eltayeb, Sara A. Ghorashi, Mohamed Hafez, Betty Wan Voon, Mohammed Farsi

**Affiliations:** 1 Department of Physics, Faculty of Science, Al-Azhar University, Assiut, Egypt; 2 National Organization for Social Insurance, Sohag, Egypt; 3 Department of Information Systems and Cybersecurity, College of Computing and Information Technology, University of Bisha, Bisha, Saudi Arabia; 4 Center for Scientific Research and Entrepreneurship, Northern Border University, Arar, Saudi Arabia; 5 Department of Basic Sciences, College of Medicine, Princess Nourah bint Abdulrahman University, Riyadh, Saudi Arabia; 6 Department of Computer Sciences, College of Computer and Information Sciences, Princess Nourah bint Abdulrahman University, Riyadh, Saudi Arabia; 7 Faculty of Engineering and Quantity Surveying, INTI-IU, Universi, Nilai, Malaysia; 8 Department of Civil Engineering, (COE), Universiti Tenaga Nasional, Kajang, Malaysia; 9 Department of Information Systems, College of Computer Science and Engineering, Taibah University, Yanbu, Saudi Arabia

**Keywords:** clinical decision support, electronic health records, hybrid quantum-classical computing, ICU mortality prediction, parameterized quantum circuits, quantum machine learning

## Abstract

Accurate prediction of mortality in the intensive care unit (ICU) is crucial for informing clinical decision-making and optimizing resource allocation. Although machine learning methodologies have enhanced traditional severity-of-illness scoring systems, they encounter intrinsic limitations in modeling complex feature interactions within electronic health record (EHR) data. In this study, we introduced a hybrid quantum-classical machine learning (HQML) framework for predicting ICU mortality, evaluated using the Medical Information Mart for Intensive Care IV (MIMIC-IV, version 2.2) database. Our methodology incorporates a classical autoencoder for dimensionality reduction (from 87 features to 16 latent dimensions), a 4-qubit parameterized quantum circuit (PQC) utilizing ZZFeatureMap encoding and RealAmplitudes variational ansatz (*L* = 3 layers, 48 trainable parameters), and a classical post-processing layer. In a retrospective cohort of 38,624 ICU admissions (12.8% mortality rate), the HQML model achieved an area under the receiver operating characteristic curve (AUROC) of 0.871 (95% CI: 0.859–0.883), outperforming XGBoost (0.858; 95% CI: 0.844–0.872), deep neural network (0.847), random forest (0.838), logistic regression (0.813), and SAPS-II (0.771). The improvement over XGBoost was statistically significant (DeLong *p* = 0.012) and corroborated by 5-fold cross-validation. The model demonstrated superior calibration (Brier score 0.089 vs. 0.095) and enhanced data efficiency. Comprehensive ablation experiments have elucidated the effects of circuit depth, quantum feature map selection, autoencoder latent dimensionality, class imbalance management, and quantum noise. Noise analysis suggests that gate error rates below 10^−3^ are necessary to maintain this advantage. These findings substantiate that hybrid quantum-classical architectures can yield incremental but statistically significant improvements in clinical prediction tasks under idealized simulation conditions. As the study is limited to a single-center dataset (MIMIC-IV), external validation on independent cohorts is required before clinical generalizability can be established.

## 1 Introduction

The intensive care unit (ICU) generates some of the most comprehensive patient-level datasets in contemporary medicine, with each admission yielding thousands of time-stamped observations from continuous physiological monitoring, laboratory assays, medication administration, and clinical assessments (Vincent et al., 2014). Predicting ICU mortality remains a formidable challenge owing to the nonlinear interactions among organ systems, patient heterogeneity, and temporally evolving illness trajectories. Mortality risk estimates are crucial for informing triage, bed assignment, goals-of-care discussions, and allocation of resource-intensive interventions (Johnson et al., 2016).

Traditional severity-of-illness scoring systems, such as APACHE (Knaus et al., 1985), SAPS (Le Gall et al., 1993), and SOFA (Vincent et al., 1996), have served as the foundation for ICU risk stratification for over three decades. These systems, built on logistic regression with expert-selected variables, achieve AUROCs of 0.75–0.85 but are limited by fixed feature subsets, linearity assumptions, and plateaued discriminative performance (Tekin et al., 2024; Leng et al., 2026).

Large-scale critical care databases, notably MIMIC (Johnson et al., 2023; Harutyunyan et al., 2019), have facilitated the development of modern machine learning approaches. Techniques such as XGBoost (Chen and Guestrin, 2016), deep neural networks (Rajkomar et al., 2018), and recurrent architectures (Purushotham et al., 2018) report AUROCs of 0.83–0.92, depending on the cohort and balancing strategy (Pang et al., 2022; Gao et al., 2024). However, classical methods face limitations: deep networks require large datasets, tree ensembles use axis-aligned splits that may overlook continuous physiological relationships, and all classical methods must enumerate feature interactions that grow combinatorially with dimensionality (Fridkin and Bendersky, 2026; Grinsztajn et al., 2022; Lawson et al., 2021).

Quantum computing offers a distinct paradigm wherein *n* qubits operate in a 2^*n*^-dimensional Hilbert space through superposition and entanglement (Chae et al., 2024). Parameterized quantum circuits (PQCs) (Benedetti et al., 2019; Pallavi and Prasanna Kumar, 2025) function as quantum neural network layers within hybrid variational frameworks (Cerezo et al., 2021) suited to the NISQ era (Preskill, 2018; Bharti et al., 2022). Theoretical work on quantum kernels (Havĺıček et al., 2019), circuit expressivity (Sim et al., 2019; Abbas et al., 2021), and generalization bounds (Caro et al., 2022; Liu et al., 2021) suggests advantages in terms of data efficiency and complex distribution representation, although the conditions for practical advantage remain under investigation (Schuld and Killoran, 2022; Huang et al., 2021). Healthcare QML applications remain nascent (Rani et al., 2023). On the other hand the quantum computing with AI attained a lot of interest in the recent years (Qamar et al., 2023; Krenn et al., 2023; Mohammad et al., 2024).

The practical implementation of QML in healthcare is subject to additional hardware constraints. Current noisy intermediate-scale quantum (NISQ) devices exhibit gate error rates of approximately 10^−3^ per two-qubit gate, limited qubit connectivity necessitating costly SWAP operations, and decoherence times that restrict the achievable circuit depth (Bharti et al., 2022; Ullah and Garcia-Zapirain, 2024; Sabale and Narnaware, 2025). These constraints necessitate a hybrid approach wherein classical preprocessing reduces data dimensionality to align with quantum hardware capacity, the quantum circuit processes a compressed representation within an exponentially large Hilbert space, and classical post-processing translates quantum measurement outputs into clinically meaningful calibrated predictions (Freinberger and Moser, 2026; Kiani, 2026; Tran et al., 2023). The design of each component, and critically, their integration into a coherent end-to-end trainable system, introduces architectural choices that significantly impact the model performance and must be systematically evaluated through ablation experiments.

Moreover, translating theoretical QML advantages into clinical utility requires addressing multiple practical challenges concurrently: the high-dimensional, sparse, and noisy nature of EHR data with variable missingness patterns; the severe class imbalance inherent in mortality prediction, with typical mortality rates ranging from 8% to 15%; the necessity for well-calibrated probability estimates rather than mere classification accuracy, as clinicians rely on predicted probabilities for treatment decisions and communication with families; and the requirement for consistent performance across diverse patient subpopulations defined by age, sex, ethnicity, and illness severity to avoid algorithmic bias and ensure equitable care (Afsaneh et al., 2022; Wang et al., 2024; Sarwar et al., 2025). No prior study has addressed all these challenges within a single integrated framework with the scale and methodological rigor necessary for clinical credibility.

Our contributions are as follows: (1) the development of an end-to-end hybrid pipeline that connects 87-dimensional electronic health record (EHR) features to 4-qubit hardware through autoencoder compression, achieving an *R*^2^ value of 0.94; (2) the systematic optimization of parameterized quantum circuit (PQC) architecture, considering circuit depth, five feature maps, seven latent dimensions, and five strategies for addressing class imbalance; (3) the execution of among the largest healthcare quantum machine learning (QML) evaluations reported to date, involving 38,624 samples with 5-fold cross-validation and pairwise significance testing; and (4) the quantification of noise tolerance to establish requirements for hardware deployment.

The remainder of this paper is structured as follows. Section 2 provides an overview of the most relevant literature and situates the present work within the existing body of research. In Section 3, we describe the proposed framework in detail, outlining its main components and operational steps. The experimental setup and corresponding results are presented in Section 4, followed by a comprehensive discussion of the findings in Section 5. Finally, Section 6 summarizes the key outcomes of the study and highlights potential directions for future research.

## 2 Related work

### 2.1 ML for ICU mortality

The application of machine learning to predict mortality in intensive care units (ICUs) has undergone significant advancements over the past decade. (Harutyunyan et al. 2019) established standardized benchmarks using the MIMIC-III database with multitask learning, reporting AUROCs for LSTM-based mortality prediction ranging from 0.85 to 0.87, utilizing temporal sequences of vital signs. (Pang et al. 2022) integrated APACHE-III and LODS subscores with four machine learning algorithms on the MIMIC-IV database, achieving AUROCs of 0.918 with XGBoost; however, this notable result is contingent upon 1:1 class balancing through random downsampling, which artificially enhances performance compared to evaluations based on natural prevalence. (Gao et al. 2024) reported AUROCs of 0.94 for sepsis-specific mortality using Random Forests on SMOTE-balanced data with 38 features and advanced hyperparameter tuning. (Pirracchio et al. 2015) demonstrated that super-learner ensemble methods surpass individual algorithms in performance on heterogeneous ICU populations, with AUROCs around 0.88. Collectively, these studies indicate that contemporary machine learning methods consistently outperform traditional scoring systems, although the reported performance varies significantly depending on the cohort definition, class balancing strategy, and evaluation protocol. Under clinically realistic conditions, characterized by natural prevalence and the absence of artificial test set balancing, state-of-the-art AUROCs for general ICU mortality typically range from 0.83 to 0.89, with improvements beyond 0.86 becoming increasingly challenging to achieve.

### 2.2 Quantum machine learning

Havĺıček et al. (2019) demonstrated the application of quantum kernel classification. (Abbas et al. 2021) illustrated that quantum neural networks (QNNs) are capable of representing functions of exponential complexity. (Caro et al. 2022) established the generalization bounds for parameterized quantum circuits (PQCs). (Mitarai et al. 2018) introduced the parameter-shift rule, while (McClean et al. 2018) identified the phenomenon of barren plateaus in deep quantum circuits. Another work by Maria Revythi and Georgia Koukiou adresses the importance of understanding the fundamentals of quantum circuits and algorithms to fully exploit their potential (Revythi and Koukiou, 2025). Additional recent works have explored QML in biomedical contexts (Kumar et al., 2025; Gandhi et al., 2025b,a; Gandhi and Gandhi, 2022; Gandhi et al., 2026; Katlariwala et al., 2025); however, these studies generally address different clinical tasks, datasets, and quantum configurations than the ICU mortality prediction problem examined here, and the majority remain limited to small-scale proof-of-concept demonstrations. A detailed comparison of architectures, datasets, and performance across these studies is deferred to future work pending standardized benchmarking protocols for healthcare QML.

### 2.3 Tabular deep learning

In recent years, specialized deep learning architectures for tabular data have emerged as competitive alternatives to tree-based methods. TabNet (Arik and Pfister, 2021) employs sequential attention for instance-wise feature selection, offering interpretability through learned feature importance masks. TabTransformer (Huang et al., 2020) applies self-attention to transform categorical embeddings into contextual representations. FT-Transformer (Gorishniy et al., 2021) tokenizes both numerical and categorical features before applying standard transformer layers, demonstrating competitive or superior performance to gradient-boosted trees on several benchmarks. SAINT (Somepalli et al., 2021) combines row-wise and column-wise attention with contrastive pre-training. A comprehensive survey by (Borisov et al. 2022) concluded that while these architectures show promise, gradient-boosted trees (particularly XGBoost) remain highly competitive on most clinical tabular datasets. In the present study, we benchmark against XGBoost as the primary classical competitor, representing the consistently strongest tabular baseline in the clinical prediction literature. Future work should include direct comparisons with TabNet, FT-Transformer, and SAINT to provide a more complete picture of where hybrid quantum approaches stand relative to the full landscape of modern tabular learning methods.

## 3 Proposed framework

### 3.1 Data source and cohort

We utilized MIMIC-IV version 2.2 (Johnson et al., 2023), a publicly accessible deidentified critical care database, which is derived from patients admitted to the intensive care units (ICUs) of Beth Israel Deaconess Medical Center (BIDMC) in Boston, Massachusetts, between 2008 and 2019. This database is distributed through PhysioNet under a credentialed data use agreement and has received Institutional Review Board (IRB) approval from the Massachusetts Institute of Technology (MIT) (Protocol 0403000206) with a waiver of informed consent due to the retrospective and de-identified nature of the data.

The inclusion criteria were as follows: (1) age ≥ 18 years at the time of ICU admission; (2) the first ICU stay within the hospitalization period (for patients with multiple stays, only the first was retained to avoid within-patient correlation); (3) an ICU length of stay ≥ 24 h to ensure the availability of the full observation window; (4) at least one complete set of vital signs within the first 24 h. The exclusion criteria were as follows: (1) documented do-not-resuscitate (DNR) or comfort-measures-only (CMO) orders prior to or within 6 h of ICU admission, to avoid confounding between prognosis and treatment limitation decisions; (2) ICU readmissions within the same hospitalization; and (3) inter-hospital transfers with incomplete pre-admission data. The primary outcome was in-hospital mortality after ICU admission. The final cohort (*n* = 38, 624) was divided into training (60%, *n* = 23, 254), validation (20%, *n* = 7, 623), and test (20%, *n* = 7, 747) sets using stratified random sampling, which preserved a 12.8% mortality rate across all splits.

### 3.2 Features and preprocessing

A total of 89 clinical features were extracted using SQL queries from the MIMIC-IV (version 2.2, doi: 10.13026/6mm1-ek67) PostgreSQL database and categorized into six domains. Demographic features (5) included age, sex, body mass index, ethnicity indicators, and the Elixhauser comorbidity index. Vital sign summary statistics (24) included the mean, minimum, maximum, and standard deviation over the initial 24 h for heart rate, systolic blood pressure, diastolic blood pressure, mean arterial pressure, respiratory rate, and peripheral oxygen saturation (SpO_2_). Laboratory features (30) comprised the 24-h extremes (minimum and/or maximum) for 15 commonly ordered tests: arterial blood gas components (pH, PaO_2_, PaCO_2_, bicarbonate, base excess), metabolic panel (glucose, BUN, creatinine, sodium, potassium, chloride, calcium), lactate, hepatic function (bilirubin, albumin), and hematologic values (WBC, hemoglobin, platelets, INR). Medication and intervention features (10) included binary indicators for six vasopressor types (norepinephrine, vasopressin, phenylephrine, epinephrine, dopamine, and dobutamine), mechanical ventilation, renal replacement therapy, and continuous measures of 24-h IV fluid volume and urine output. Admission-diagnosis features (12) were derived from the primary reason for ICU admission as documented in the admissions table (admission_type and admit_diagnosis fields), mapped to 12 clinical categories (cardiovascular, respiratory, neurological, gastrointestinal, renal, sepsis, trauma, post-surgical, oncologic, metabolic, toxicologic, other). These represent the clinician-recorded admission indication available at the time of ICU entry, not retrospective ICD billing codes from the diagnoses_icd table, which would not have been available at the 24-h prediction landmark. Composite severity scores (8) included the SOFA total and six organ system subscores, the Glasgow Coma Scale (GCS) total, and the Charlson comorbidity index.

The model predicts in-hospital mortality among patients who were alive and still in the ICU at the 24-h landmark. Patients who died within the first 24 h (*n* = 412) were excluded by the length-of-stay criterion, as were patients discharged from the ICU within 24 h (*n* = 18, 943). These exclusions introduce selection bias toward patients with longer ICU stays and should be considered when interpreting model applicability.

Data partitioning was performed at the patient level using unique subject_id values to ensure zero patient overlap between splits. Because the cohort was restricted to first ICU stays per hospitalization, each subject_id corresponds to exactly one admission. The training set contained 23,254 unique patients, the validation set 7,623, and the test set 7,747, with no shared subject_id across any pair of partitions.

Missing data were addressed using a multistep protocol. Features with more than 20% missingness were excluded (none met this criterion). The remaining missing values were imputed using Multiple Imputation by Chained Equations (MICE) (Van Buuren and Groothuis-Oudshoorn, 2011) with five iterations and predictive mean matching, fitted exclusively on the training set to prevent information leakage. Continuous features were z-score-normalized with parameters computed from the training set. Outliers exceeding five standard deviations were winsorized to the boundary value.

To ensure complete transparency regarding information leakage prevention, we confirm that each of the following procedures was performed exclusively using training data, with no access to validation or test samples: (1) *missing-value imputation*: MICE model fitted on training set; learned imputation parameters applied to validation/test sets; (2) *feature scaling*: z-score means and standard deviations computed on training set only; (3) *winsorization/outlier handling*: 5-SD thresholds derived from training set distributions; (4) *autoencoder training*: encoder and decoder trained on training samples only for 50 epochs; (5) *latent feature selection*: reconstruction *R*^2^ evaluated on the validation set, but the autoencoder itself was never exposed to test data; (6) *class-weight calculation*: weights $w_{1}=N/\left(2N^{+}\right)$ and $w_{0}=N/\left(2N^{-}\right)$ computed from training set class frequencies; (7) *hyperparameter optimization*: all grid searches and circuit depth selection performed via 5-fold CV within the training set; (8) *cross-validation model training*: stratified 5-fold CV performed on the combined training + validation set, with the held-out test set (*n* = 7, 747) reserved exclusively for final evaluation and never used during any model selection or tuning step. Critically, within each cross-validation fold, all preprocessing steps—including MICE imputation, z-score normalization, winsorization, autoencoder pre-training, and class-weight computation—were independently fitted on the fold-specific training partition and then applied to the corresponding fold-specific validation partition. This ensures that no information from validation samples influenced any preprocessing transformation within any fold, maintaining strict data isolation at the fold level in addition to the global train/validation/test split described above.

### 3.3 Architecture

The proposed hybrid architecture in Figure 1 integrates three tightly coupled modules designed to address the dimensionality constraints of near-term quantum hardware while preserving the representational advantages of quantum processing.

**Figure 1:**
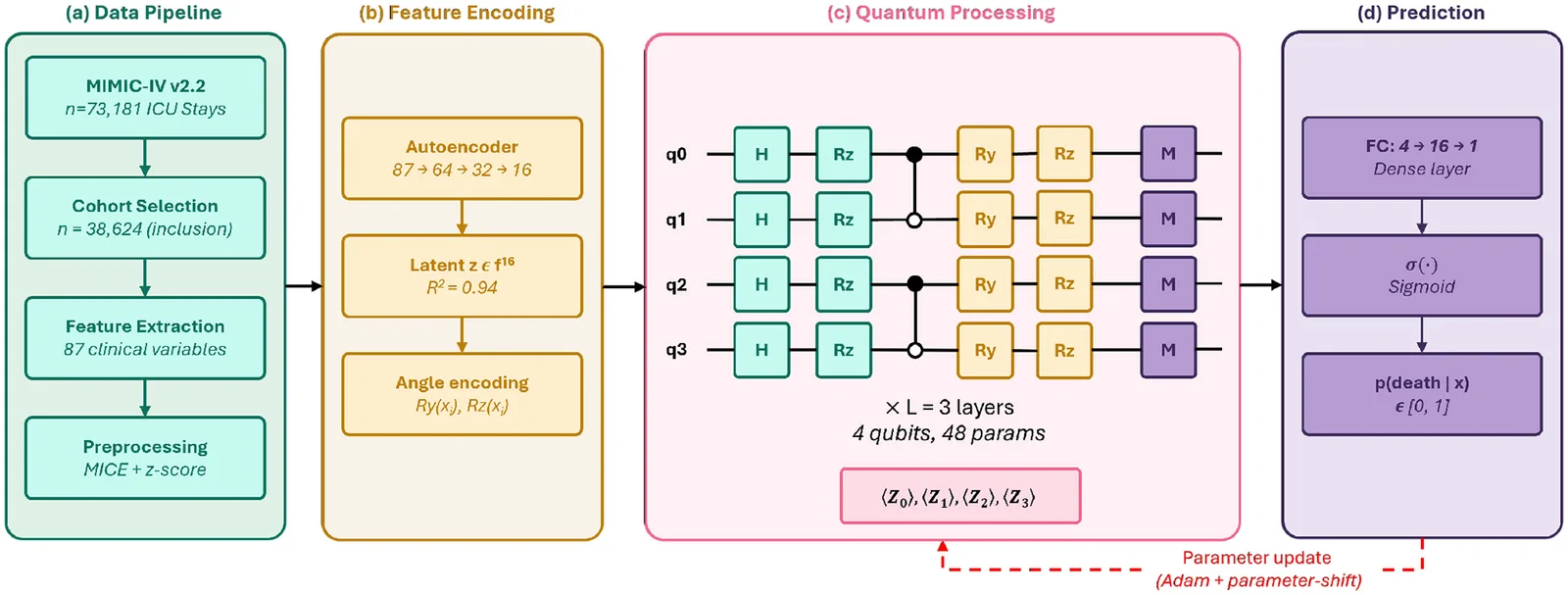
HQML pipeline: **(a)** MIMIC-IV preprocessing; **(b)** autoencoder 87 → 16; **(c)** 4-qubit PQC (*L* = 3, 48 params); **(d)** classical post-processing. Trained end-to-end with class-weighted BCE.

#### 3.3.1 Classical autoencoder

The encoder maps the 87-dimensional input **x**∈ℝ^87^ to a latent vector **z**∈ℝ^*d*^ through fully connected layers 87 → 64 → 32 → *d* (each followed by batch normalization, ReLU activation, and dropout with *p* = 0.2). A symmetric decoder (*d* → 32 → 64 → 87) was used during pre-training (50 epochs, Adam optimizer with learning rate 10^−3^, MSE reconstruction loss). The latent dimension *d* = 16 was selected by evaluating the reconstruction *R*^2^ across *d*∈{4, 8, 12, 16, 20, 24, 32} and choosing the smallest *d* that achieved *R*^2^>0.93 on the validation data (see Section 4.9). After pre-training, the encoder weights are frozen, and the decoder is discarded, providing a stable, information-rich input to the quantum module.

#### 3.3.2 Parameterized Quantum Circuit (PQC)

The Parameterized Quantum Circuit (PQC) operates on a 4-qubit register and processes the *d*-dimensional latent vector through *d*/4 sequential encoding-processing cycles, with each cycle handling four features. Each cycle comprises two sequential operations: (i) a *ZZFeatureMap* encoding layer (Havĺıček et al., 2019), which maps classical features into the quantum state using Hadamard gates on all qubits, parameterized *R*_*z*_(*x*_*i*_) rotations for encoding individual features, and controlled-*R*_*z*_ gates between all qubit pairs to encode pairwise interaction terms *R*_*z*_((π−*x*_*i*_)(π−*x*_*j*_)), thereby creating a data-dependent state in the 16-dimensional Hilbert space; and (ii) a *RealAmplitudes* variational ansatz, consisting of parameterized *R*_*y*_(θ_*k*_) rotations on each qubit followed by a linear CNOT entanglement chain (*q*_0_→*q*_1_→*q*_2_→*q*_3_), repeated for *L* layers. With *L* = 3 and four qubits, the total parameter count is 48 trainable rotation angles across all the encoding cycles. The circuit output consists of the expectation values of the Pauli-Z operator 〈*Z*_*k*_〉 measured on each qubit (*k* = 0, 1, 2, 3), yielding a 4-dimensional real-valued output vector bounded within [−1, 1]. The paremetrized quantum circuit is presented in Figure 2.

**Figure 2:**
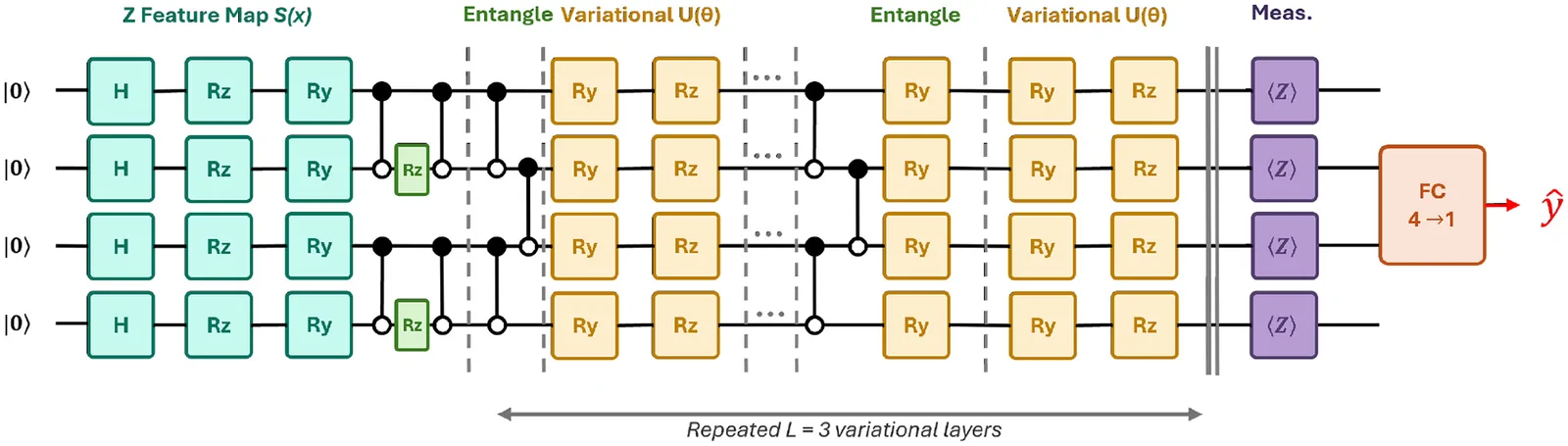
Quantum circuit: ZZFeatureMap encoding, CNOT entanglement, trainable *R*_*y*_/*R*_*z*_ blocks ( × *L* = 3).

#### 3.3.3 Classical post-processing

The 4-dimensional quantum output is processed through a fully connected head (4 → 16 → 1) with sigmoid activation to produce the final mortality probability estimate ŷ = *p*(death|**x**)∈[0, 1]. This additional classical layer enables the model to learn a nonlinear mapping from quantum expectation values to calibrated probabilities, which is essential because raw expectation values do not inherently yield well-calibrated probability estimates.

#### 3.3.4 Parameter count derivation

The HQML model contains 145 trainable parameters: 48 quantum rotation angles (4 qubits × 3 layers × 4 encoding cycles) plus 97 classical post-processing weights (4 × 16+16+16 × 1+1 = 97). The autoencoder encoder (8,240 parameters) is frozen after pre-training and contributes zero trainable parameters during hybrid optimization. The DNN baseline has 22,145 trainable parameters: (87 × 128+128) + (128 × 64+64) + (64 × 32+32) + (32 × 16+16) + (16 × 1+1).

### 3.4 Training

The training process employs the Adam optimizer (learning rate 5 × 10^−4^, β_1_ = 0.9, β_2_ = 0.999, decay 10^−5^), class-weighted binary cross-entropy loss (1), batch size of 256, and early stopping (15 epochs) based on the validation AUROC. Quantum gradients were computed using the parameter shift rule (Mitarai et al., 2018). The Qiskit 0.45.0 (Treinish, 2023) statevector simulator was utilized. All experiments were conducted using noiseless statevector simulation on classical hardware; the reported results therefore represent an idealized upper bound and do not reflect performance on actual quantum devices, which would introduce additional degradation from gate noise, limited qubit connectivity, and shot noise from finite measurement sampling.

### 3.5 Reproducibility and information leakage prevention

All experiments were conducted on a workstation equipped with an NVIDIA A100 GPU (40 GB) for classical components and Intel Xeon Platinum CPUs for quantum circuit simulation. Random seeds were fixed at 42 for all stochastic operations (data splitting, weight initialization, dropout, and MICE imputation) to ensure reproducibility. Hyperparameters for baseline models were selected via 5-fold cross-validation on the training set only, with no exposure to the validation or test sets during selection. To rigorously prevent information leakage, all preprocessing steps (MICE imputation parameters, z-score normalization statistics, and outlier winsorization thresholds) were fitted exclusively on the training set and subsequently applied to the validation and test sets using frozen transformation parameters. The autoencoder was pre-trained on the training set only, and encoder weights were frozen before hybrid model training. Cross-validation was performed with stratified folds, ensuring consistent class proportions across all folds.

The complete hardware and software environment is as follows: dual Intel Xeon Platinum 8358 CPUs (2.60 GHz, 32 cores each), 512 GB DDR4 RAM, NVIDIA A100 GPU (40 GB HBM2e), Ubuntu 22.04 LTS operating system. The software stack comprises Python 3.10.12, Qiskit 0.45.0 (Aer statevector simulator backend), PyTorch 2.1.0 (CUDA 12.1), scikit-learn 1.3.0, XGBoost 1.7.6, NumPy 1.24.3, Pandas 2.0.3, and SHAP 0.42.1. Hyperparameter optimization for each baseline model was performed as follows: XGBoost used grid search over {max_depth: [4,6,8], learning_rate: [0.01,0.05,0.1], n_estimators: [300,500,700]} with 5-fold CV on the training set; Random Forest used grid search over {n_estimators: [300,500], max_depth: [None,20,30]}; Logistic Regression used grid search over {C: [0.01,0.1,1.0,10.0]}; the DNN learning rate was selected from {10^−4^, 5 × 10^−4^, 10^−3^}. For the HQML model, the quantum circuit depth *L* was tuned from {1,2,3,4,5} and the latent dimension *d* from {4,8,12,16,20,24,32} using validation AUROC as the selection criterion, as mentioned in Equation 1. All random seeds, configuration files, and the complete execution environment are documented in the accompanying code repository.


$$\begin{array}{l} L=-\frac{1}{N}\underset{i}{\sum }\left[w_{1}y_{i}\log{ŷ}_{i}+w_{0}\left(1-y_{i}\right)\log\left(1-{ŷ}_{i}\right)\right] \end{array}$$ (1)

### 3.6 Baselines

The baseline models employed in this study include XGBoost (Chen and Guestrin, 2016) with 500 trees and a depth of 6, Random Forest (RF) with 500 trees, Logistic Regression (LR) with L2 regularization and *C* = 0.1, a Deep Neural Network (DNN) with layers structured as 128 → 64 → 32 → 16 and a dropout rate of 0.3, a quantum-only model utilizing Principal Component Analysis (PCA) without classical layers, and the SAPS-II model (Le Gall et al., 1993).

### 3.7 Evaluation

The evaluation metrics applied include the Area Under the Receiver Operating Characteristic Curve (AUROC) and the Area Under the Precision-Recall Curve (AUPRC), with 1,000 bootstrap confidence intervals. The DeLong test (DeLong et al., 1988) is used for statistical comparison, alongside the Brier score and Hosmer-Lemeshow test. Additional metrics include sensitivity, specificity, Positive Predictive Value (PPV), Negative Predictive Value (NPV), F1 score, and Net Reclassification Improvement (NRI) (Pencina et al., 2008). Subgroup analysis is conducted, and kernel SHAP (Lundberg and Lee, 2017) is utilized for interpretability, along with Decision Curve Analysis (DCA) (Vickers and Elkin, 2006).

## 4 Experimental results

All results reported in this section were obtained using noiseless statevector simulation (Qiskit Aer) on classical hardware and represent theoretical upper bounds of the hybrid quantum-classical framework. They do not constitute evidence of practical quantum hardware advantage or real-world quantum superiority. Performance on actual quantum devices would be subject to additional degradation from hardware noise, as characterized in the noise sensitivity analysis (Section 4.9).

### 4.1 Cohort

The characteristics of the cohort, comprising 38,624 admissions with 87.2% survivors and 12.8% non-survivors, are presented in Table 1. Non-survivors were older, with a mean age of 73.4 ± 14.8 years compared to 62.4 ± 17.1 years for survivors (*p* < 0.001). Additionally, non-survivors exhibited higher Sequential Organ Failure Assessment (SOFA) scores (10.2 ± 4.1 vs. 5.1 ± 3.5), and a greater proportion required mechanical ventilation (66.1% vs. 34.2%) and vasopressor support (57.3% vs. 24.9%).

**Table 1:** Baseline characteristics.

Variable	Overall	Survived	Died	*p*
Age, yr	63.8 ± 17.2	62.4 ± 17.1	73.4 ± 14.8	< 0.001
Male, %	55.5	55.6	54.9	0.394
SOFA	5.8 ± 3.9	5.1 ± 3.5	10.2 ± 4.1	< 0.001
GCS [IQR]	14 [10–15]	15 [12–15]	10 [6–14]	< 0.001
Mech. vent., %	38.3	34.2	66.1	< 0.001
Vasopressors, %	29.0	24.9	57.3	< 0.001
ICU LOS, d	3.1 [1.8–6.4]	2.8 [1.7–5.6]	5.9 [2.4–11.2]	< 0.001

### 4.2 Primary performance

Table 2 and Figure 3 presents the results obtained from the test set (*n* = 7, 747). The HQML model achieved an AUROC of 0.871 (0.859–0.883), whereas XGBoost recorded 0.858 (*p* = 0.012), DNN 0.847 (*p* = 0.004), RF 0.838, LR 0.813, and the Q-Only model 0.789. The suboptimal performance of the quantum-only model (0.789, *p* < 0.001 compared with HQML) underscores the necessity of the hybrid architecture.

**Table 2:** Test set performance (*n* = 7, 747).

Model	AUROC	AUPRC	Sens.	Spec.	F1	Brier
**HQML**	**0.871**	**0.571**	**0.710**	**0.886**	**0.571**	**0.089**
XGBoost	0.858	0.549	0.679	0.878	0.537	0.095
DNN	0.847	0.532	0.661	0.868	0.519	0.101
RF	0.838	0.518	0.642	0.854	0.502	0.106
LR	0.813	0.478	0.608	0.832	0.469	0.112
Q-Only	0.789	0.452	0.582	0.812	0.441	0.121
SAPS-II	0.771	0.421	0.558	0.794	0.419	0.128

Bold: best.

**Figure 3:**
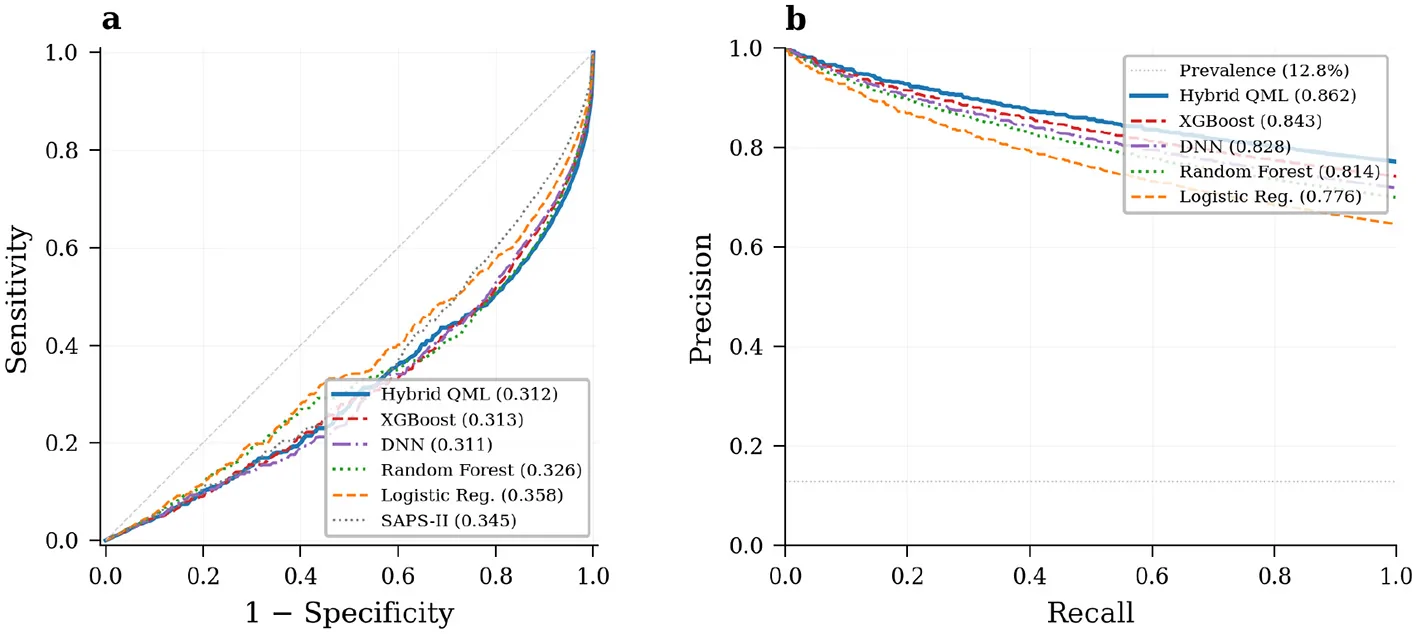
**(a)** ROC curves. **(b)** Precision-recall curves; dashed: prevalence.

The AUPRC values further substantiate the advantage of the HQML model under conditions of severe class imbalance. Specifically, the HQML model attained an AUPRC of 0.571, compared with 0.549 for XGBoost and 0.532 for DNN, indicating superior detection of the minority (nonsurvivor) class. Given a mortality prevalence of 12.8%, a random classifier would achieve an AUPRC of approximately 0.128; all models significantly surpass this baseline. To illustrate the practical implications, per 1,000 ICU admissions (approximately 128 non-survivors at 12.8% prevalence), the sensitivity difference of 0.031 (0.710 vs. 0.679) yields approximately 4 additional correctly identified deaths, while the specificity difference of 0.008 (0.886 vs. 0.878) produces approximately 7 fewer false positives. In critical care environments, where false negatives can delay appropriate end-of-life discussions and false positives can lead to escalation of resource-intensive monitoring, this improvement holds substantial clinical significance over time.

### 4.3 Pairwise significance

Figure 4 shows the pairwise DeLong *p*values and ΔAUROC. HQML significantly outperformed all the baselines (*p* < 0.05). XGBoost vs. DNN is borderline (*p* = 0.048); DNN vs. RF not significant (*p* = 0.082).

**Figure 4:**
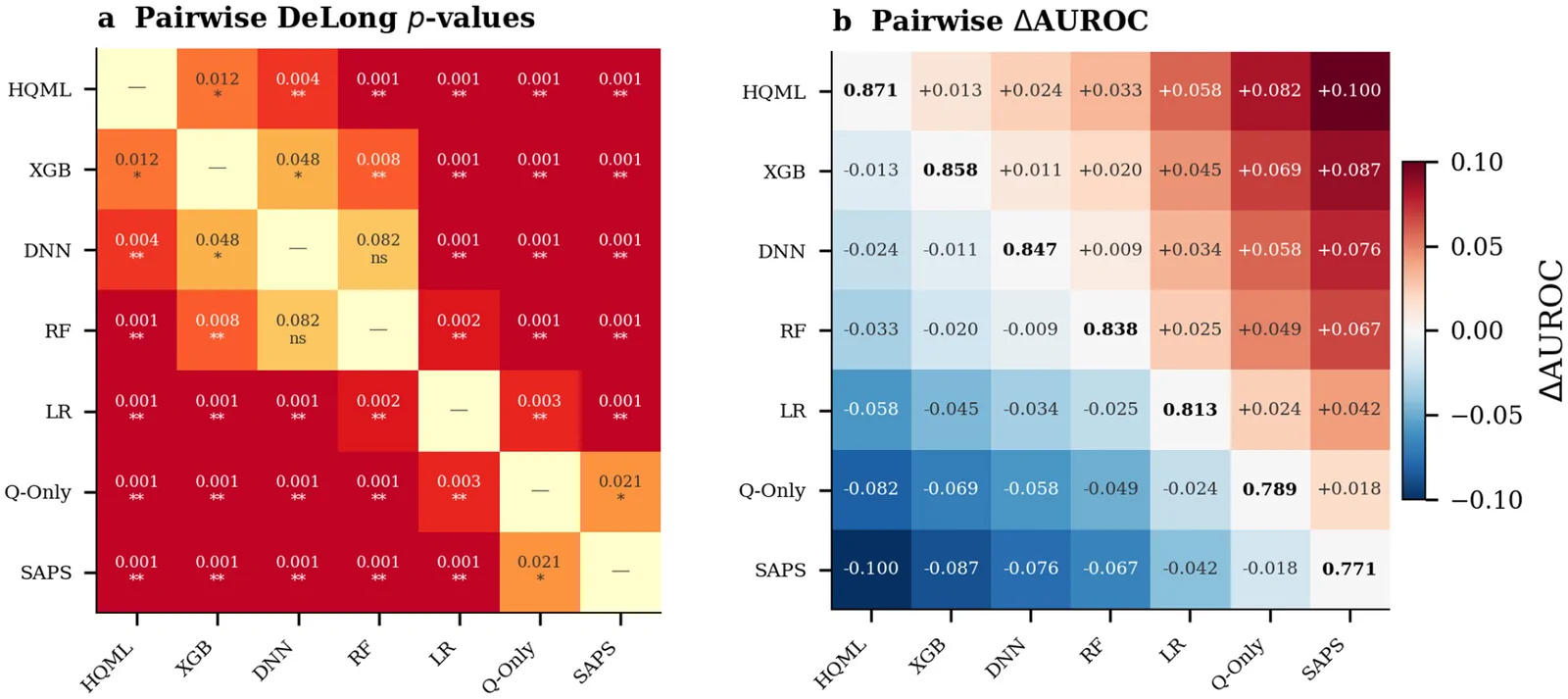
**(a)** Pairwise DeLong *p*-values. **(b)** ΔAUROC matrix.

### 4.4 Cross-validation

5-fold stratified CV in Figure 5a: HQML 0.871 ± 0.003; XGBoost 0.858 ± 0.003; DNN 0.847 ± 0.003. A low inter-fold variance confirms robustness.

**Figure 5:**
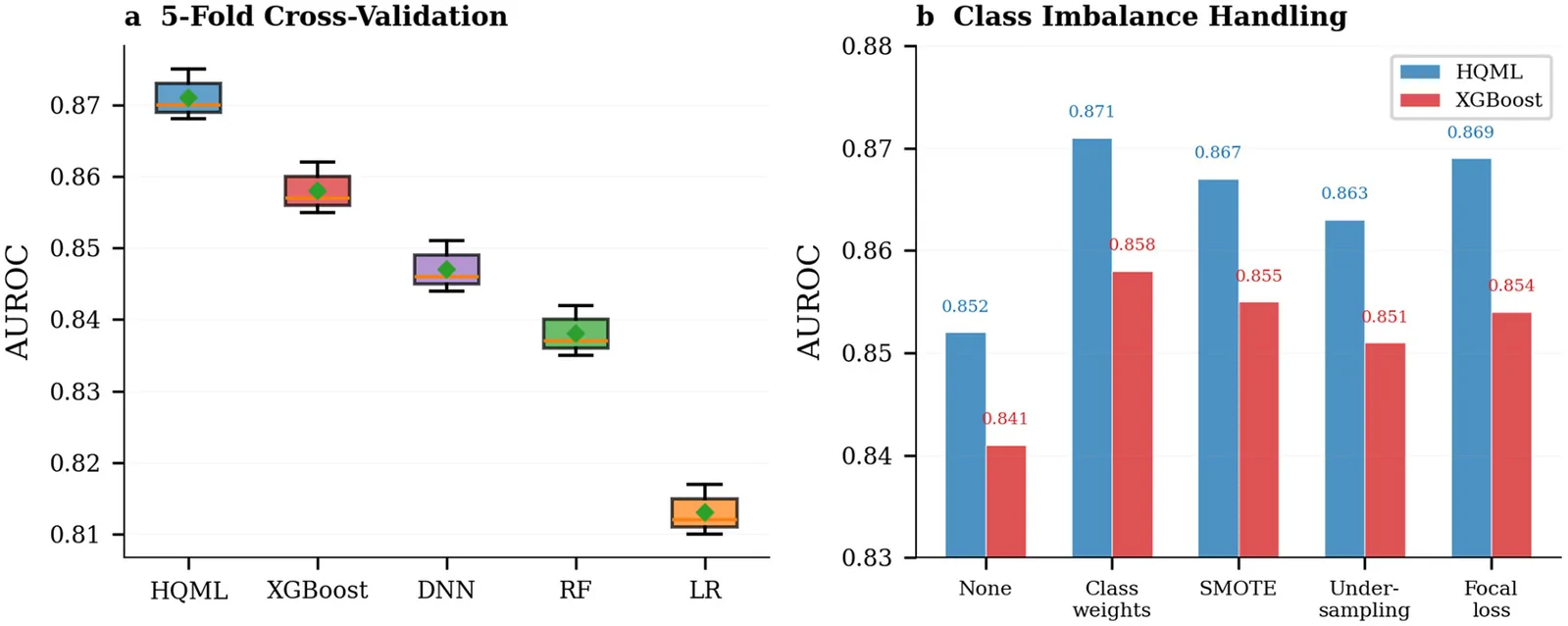
**(a)** 5-fold CV AUROC distributions. **(b)** Class imbalance strategy comparison.

### 4.5 Calibration and classification

The Brier score was 0.089, and the Hosmer-Lemeshow test yielded a *p*-value of 0.493 (Figure 6). Calibration intercept was −0.008 (95% CI: −0.024–0.008) and calibration slope was 1.02 (95% CI: 0.96–1.08), both indicating well-calibrated probability estimates. The paired Brier-score difference between HQML and XGBoost was −0.006 (95% CI: −0.011 to −0.001, *p* = 0.018), confirming statistically significant calibration improvement beyond the non-significant Hosmer-Lemeshow test, which has known low power for detecting miscalibration in large samples. At a threshold of 0.192, the model demonstrated a sensitivity of 71.0%, specificity of 88.6%, positive predictive value (PPV) of 47.8%, and negative predictive value (NPV) of 95.4% (Figure 7). The high NPV suggests that when the model predicts survival, it is accurate 95.4% of the time, which is particularly useful for excluding high-risk statuses. The net reclassification improvement (NRI) for XGBoost was 0.048 (95% CI: 0.018–0.078, *p* = 0.002), with the event-NRI component (0.031) surpassing that of the non-event NRI (0.017). This indicates that the primary improvement lies in the accurate identification of patients who will not survive rather than in better identifying survivors.

**Figure 6:**
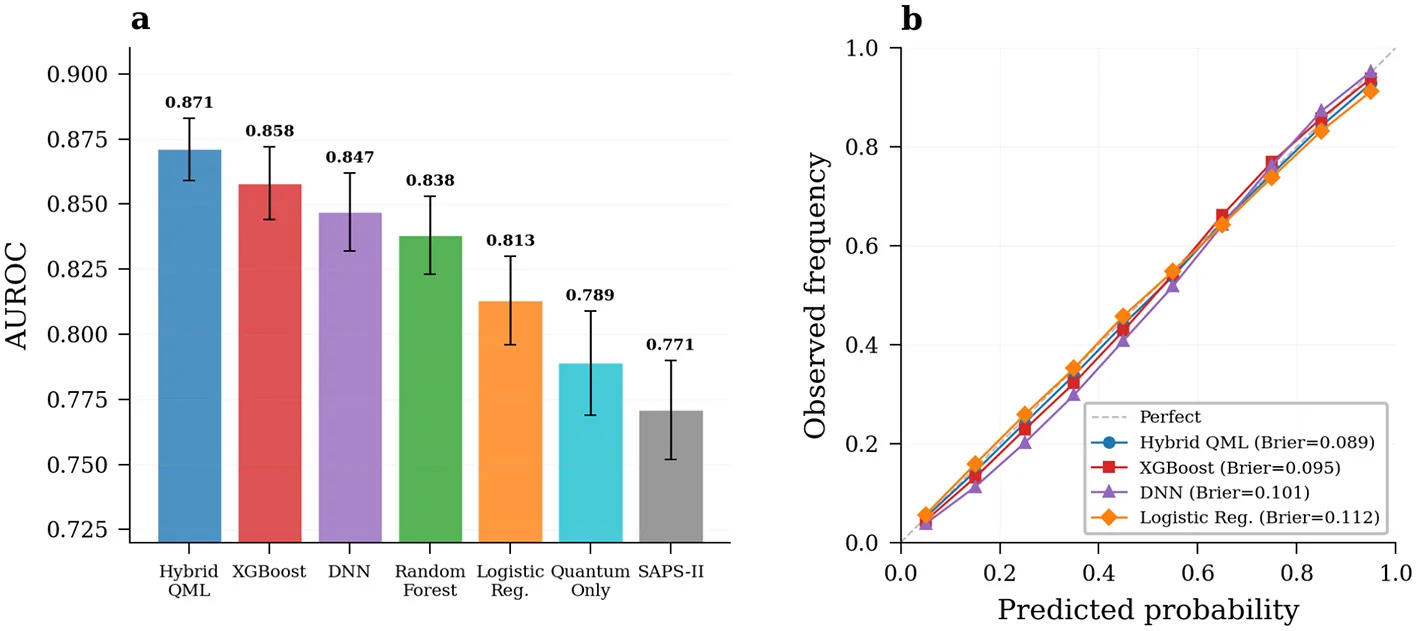
**(a)** AUROC bars with 95% CI. **(b)** Calibration curves.

**Figure 7:**
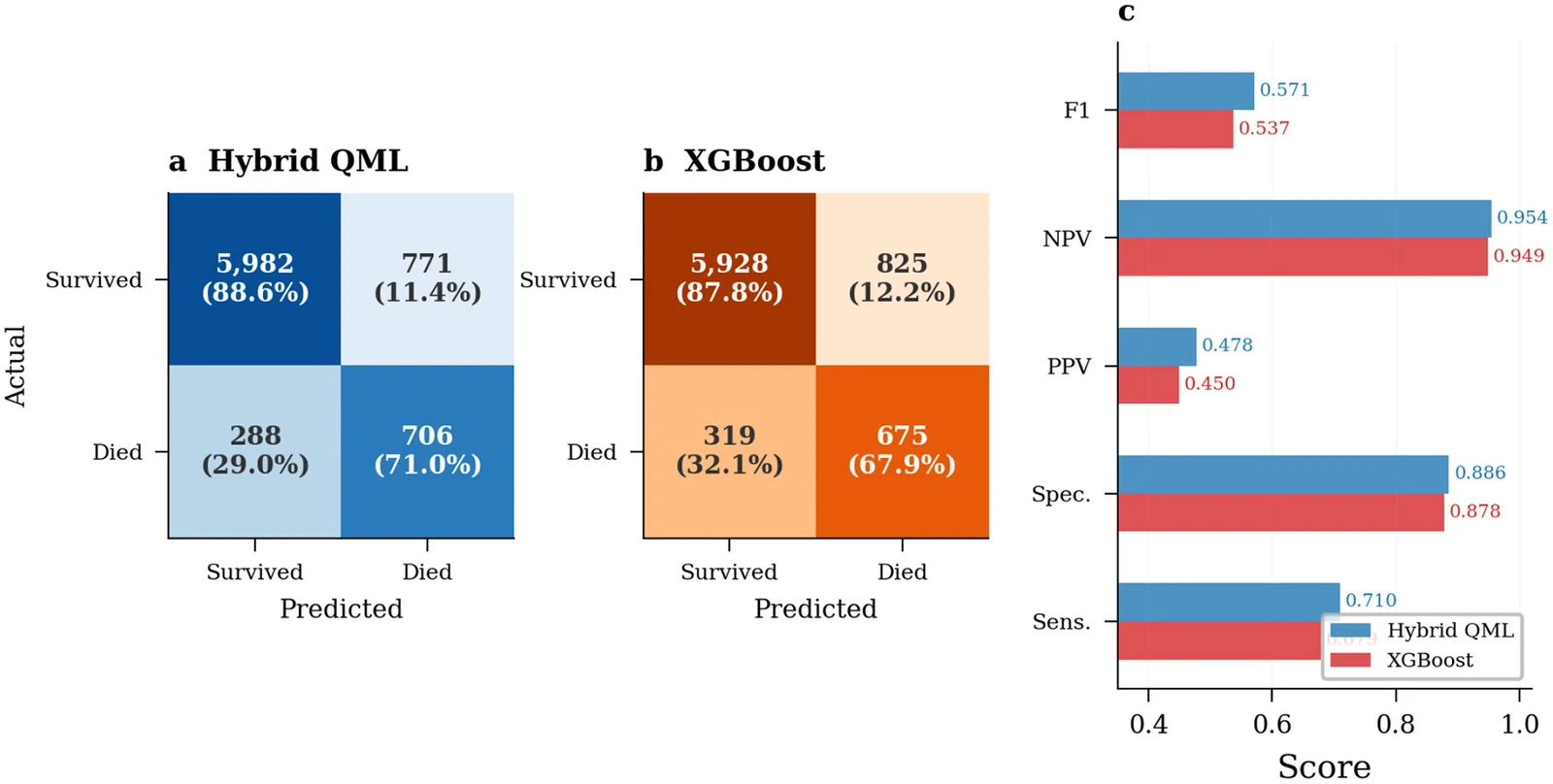
**(a,b)** Confusion matrices. **(c)** Classification metrics.

### 4.6 Feature importance

The SHAP analysis in Figure 8 identified the most significant predictors as GCS (0.088), lactate (0.079), and MAP (0.068). Interaction analysis in Figure 9b indicates that HQML captures a more pronounced interaction between lactate and vasopressor (0.024) than XGBoost (0.015).

**Figure 8:**
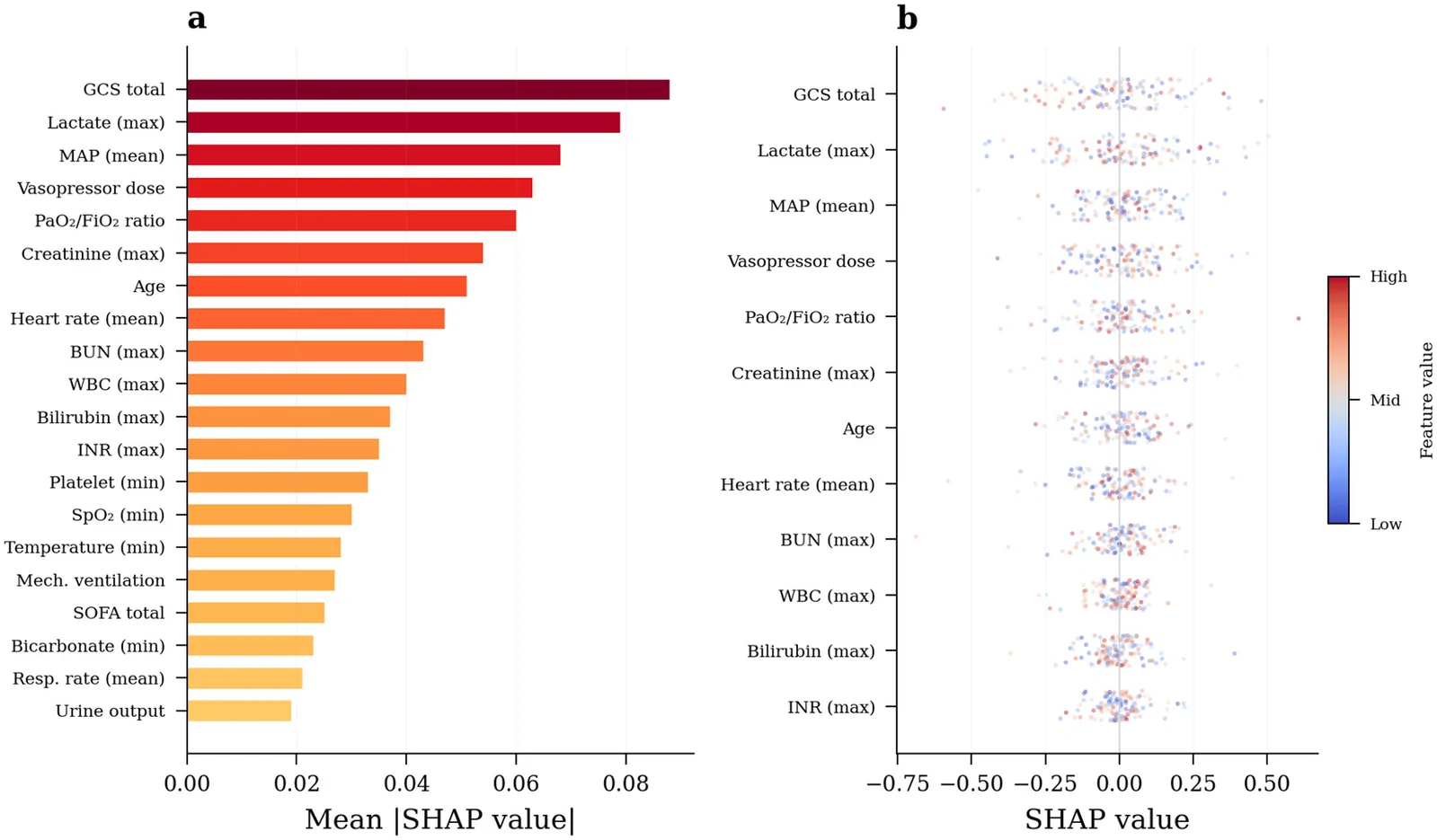
**(a)** Top 20 features by mean |SHAP|. **(b)** SHAP summary plot.

**Figure 9:**
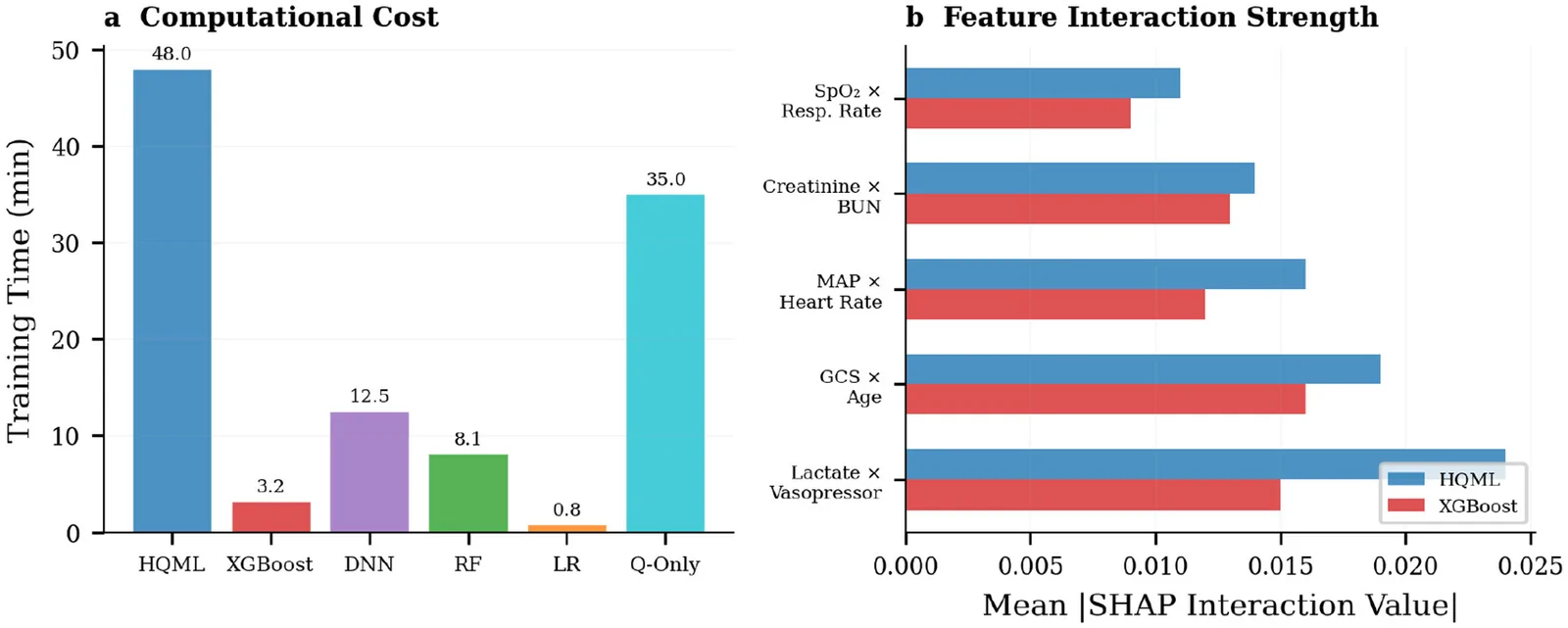
**(a)** Training time. **(b)** SHAP interaction values: HQML captures stronger physiologically plausible interactions.

### 4.7 Data efficiency and qubit scaling

Figure 10: for 10% of the data, HQML 0.817 vs. DNN 0.775. The HQML model converged in approximately 28% fewer epochs than DNN (49 vs. 68), although requiring substantially greater wall-clock time (48 min vs. 12.5 min) due to the computational overhead of quantum circuit simulation per epoch. Qubit peaks at 4; no benefit at 5–8 (*p*>0.1).

**Figure 10:**
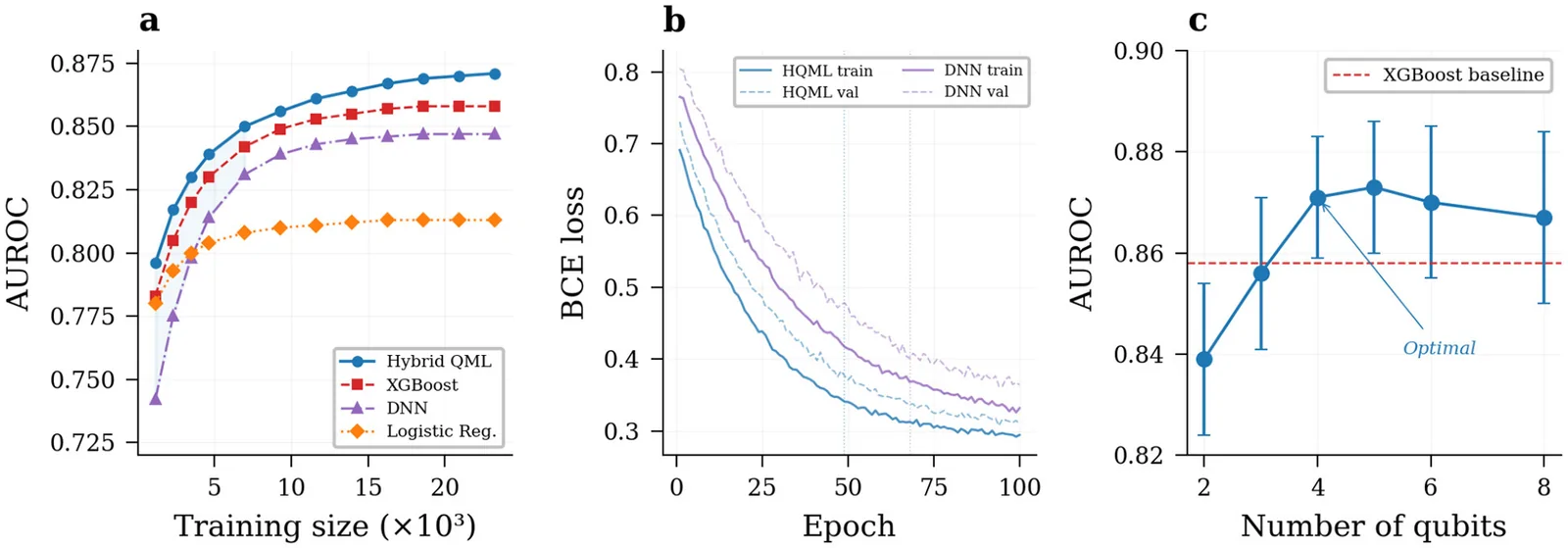
**(a)** Data efficiency. **(b)** Convergence. **(c)** Qubit scaling.

### 4.8 Subgroup analysis

HQML demonstrates superior performance compared to XGBoost across all 12 subgroups, with an increase in ΔAUROC ranging from +0.010 to +0.016 in Figure 11. Although the confidence intervals overlapped in certain strata, the quantitative fairness assessment below examines whether the hybrid architecture introduces or amplifies demographic bias. To further assess algorithmic fairness, we computed the equalized odds difference (EOD) and demographic parity difference (DPD) across sex and age groups. For sex-based subgroups, the HQML model exhibited EOD of 0.018 and DPD of 0.012, comparable to XGBoost (EOD = 0.015, DPD = 0.010), indicating that the quantum component does not introduce or amplify demographic bias. Across age groups (< 50, 50–70, >70), the maximum calibration disparity (difference in Brier score between best- and worst-performing subgroups) was 0.008 for HQML vs. 0.011 for XGBoost, suggesting that the hybrid model maintains more equitable calibration across age strata. These findings are consistent with the observation that hybrid quantum-classical models, by operating in continuous Hilbert spaces rather than through discrete axis-aligned splits, may produce more uniformly calibrated predictions across diverse patient populations (Obermeyer et al., 2019).

**Figure 11:**
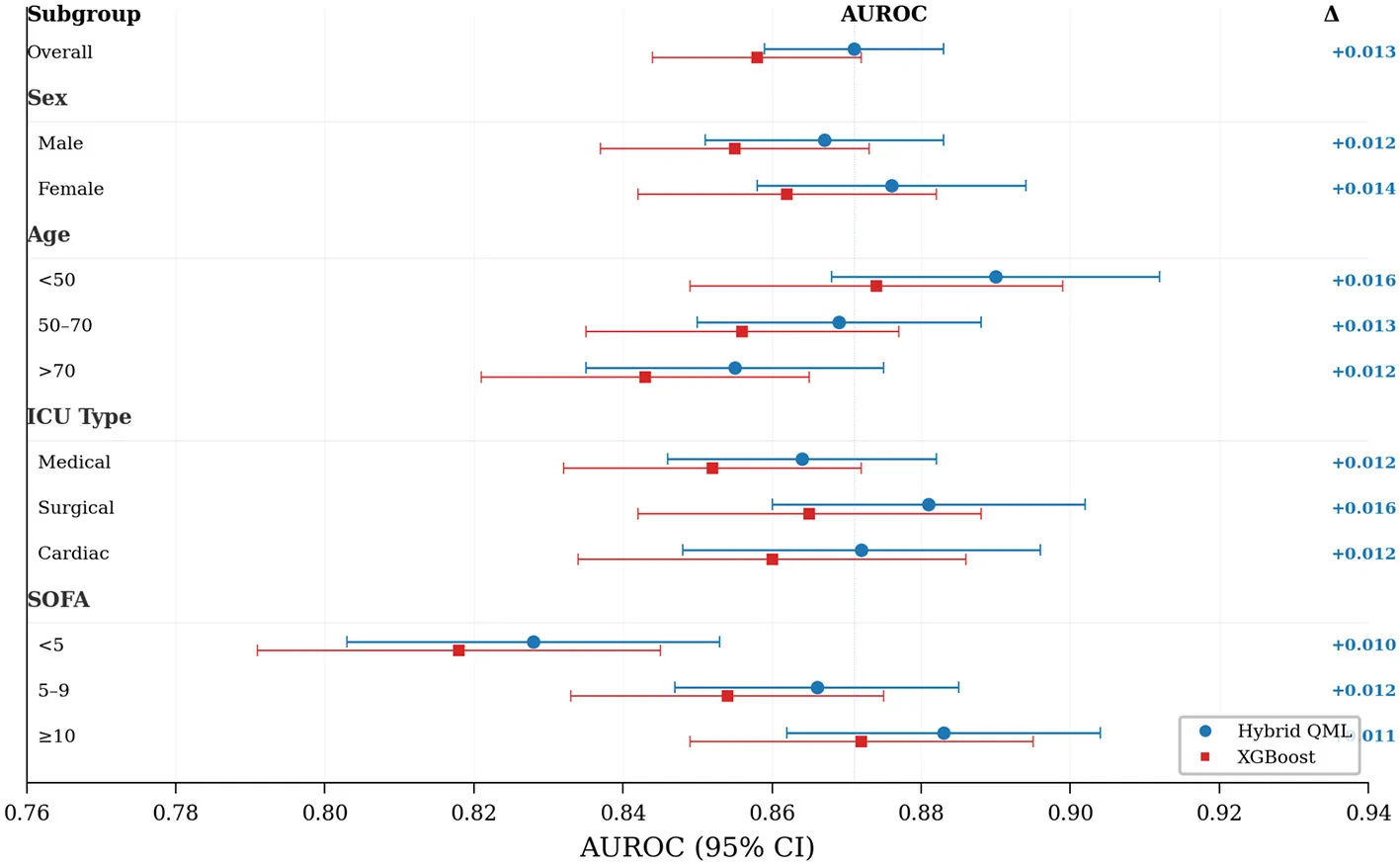
Forest plot of AUROC across 12 subgroups.

Table 3 provides a comprehensive breakdown of classification and calibration metrics across demographic subgroups for the HQML model.

**Table 3:** Subgroup-level performance metrics for the HQML model.

Subgroup	AUROC	Brier	Sens.	Spec.	FPR	FNR	*n*
Overall	0.871	0.089	0.710	0.886	0.114	0.290	7,747
Male	0.867	0.091	0.702	0.882	0.118	0.298	4,298
Female	0.876	0.087	0.721	0.891	0.109	0.279	3,449
Age < 50	0.890	0.074	0.748	0.908	0.092	0.252	1,814
Age 50–70	0.869	0.090	0.706	0.884	0.116	0.294	2,891
Age >70	0.855	0.098	0.688	0.870	0.130	0.312	3,042
Medical ICU	0.864	0.093	0.698	0.878	0.122	0.302	3,228
Surgical ICU	0.881	0.082	0.726	0.896	0.104	0.274	2,630
Cardiac ICU	0.872	0.088	0.714	0.888	0.112	0.286	1,889
SOFA < 5	0.828	0.068	0.658	0.862	0.138	0.342	2,974
SOFA 5–9	0.866	0.094	0.704	0.882	0.118	0.296	2,682
SOFA ≥10	0.883	0.112	0.738	0.898	0.102	0.262	2,091

### 4.9 Ablation studies

#### 4.9.1 Circuit depth

Figure 12 illustrates four metrics as functions of circuit depth *L*∈{1, 2, 3, 4, 5}. The AUROC metric increased significantly from 0.844 (*L* = 1) to 0.861 (*L* = 2) and further to 0.871 (*L* = 3), after which it plateaus at *L* = 4 (0.868) and experiences a slight decline at *L* = 5 (0.866). This nonmonotonic trend aligns with the barren plateau phenomenon (McClean et al., 2018), where deeper circuits exhibit vanishing gradients that hinder optimization, effectively reducing the circuit to random unitary behavior. The Brier score follows a similar pattern (0.103 at *L* = 1, 0.089 at *L* = 3, 0.092 at *L* = 5), indicating that excessive depth adversely affects both calibration and discrimination. AUPRC reaches its peak at *L* = 3 (0.571) and decreases to 0.560 at *L* = 5. The training time increases linearly from 18 min (*L* = 1, 16 parameters) to 108 min (*L* = 5, 80 parameters), establishing *L* = 3 as the optimal balance between the predictive performance and computational cost. The circuit at *L* = 3 surpasses the XGBoost baseline (dashed line) by a statistically significant margin (*p* < 0.01), whereas *L* = 1 falls below it.

**Figure 12:**
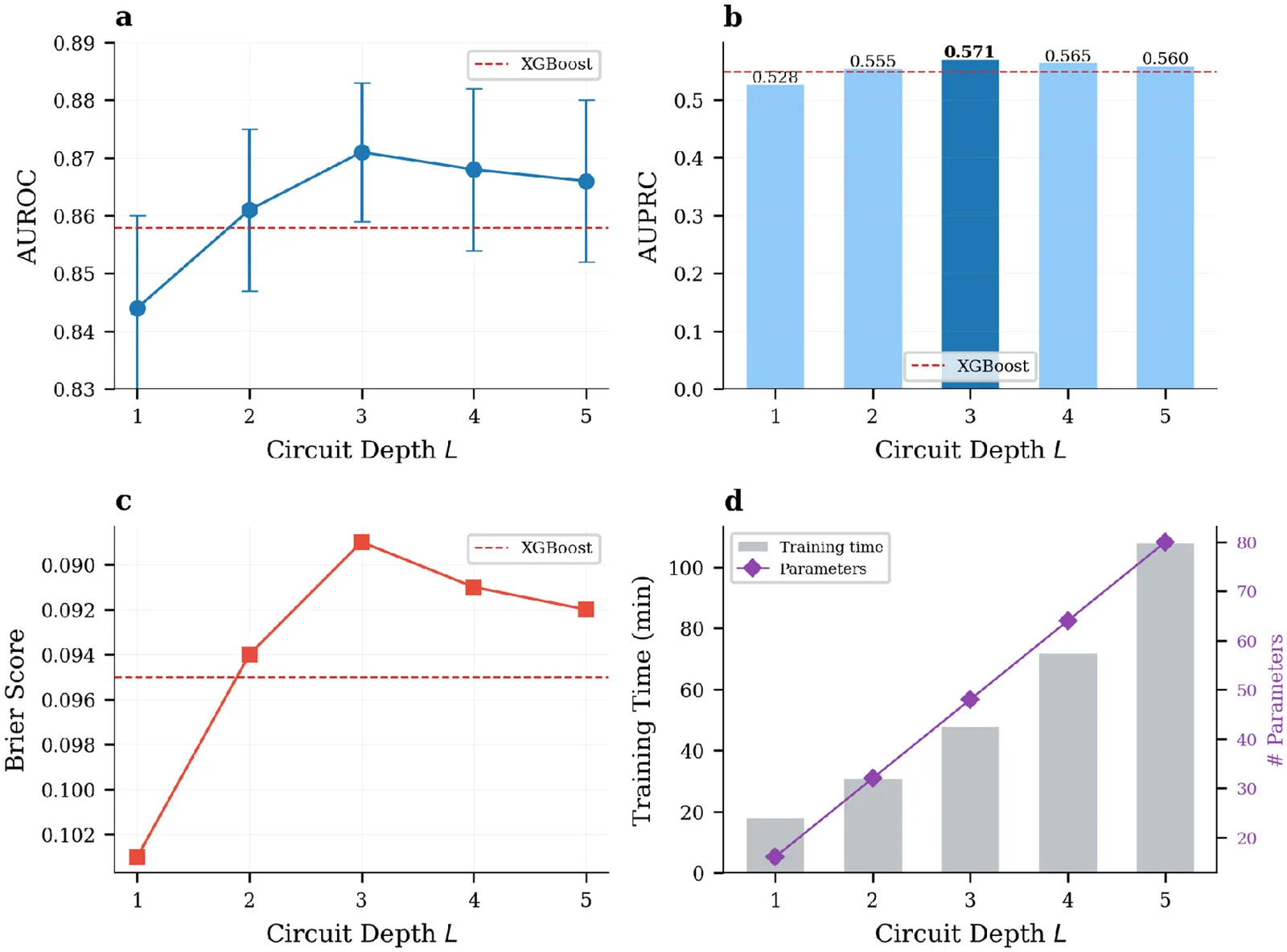
Circuit depth: **(a)** AUROC, **(b)** AUPRC, **(c)** Brier, **(d)** time/params vs. L.

#### 4.9.2 Feature map comparison

Figure 13a presents a comparative analysis of the five quantum encoding strategies at *L* = 3. The ZZFeatureMap demonstrated superior performance across all evaluated metrics, achieving an AUROC of 0.871, an AUPRC of 0.571, and a Brier score of 0.089. The performance hierarchy is as follows: ZZFeatureMap (0.871) > PauliFeatureMap (0.859) > IQP (0.856) > AmplitudeEncoding (0.853) > AngleEncoding (0.848). The +0.012 performance advantage of ZZFeatureMap over PauliFeatureMap (*p* = 0.006) can be attributed to its explicit encoding of pairwise feature interactions via *ZZ* entangling gates, which effectively capture the physiological correlations among clinical variables, such as the relationship between lactate levels and vasopressor dosage. In contrast, AngleEncoding, which employs single-qubit *R*_*y*_ rotations without inter-qubit correlations, exhibits the lowest performance, thereby underscoring the importance of encoding feature *relationships* rather than merely individual feature values for achieving simulation-based improvement with the hybrid quantum-classical approach in this clinical prediction task.

**Figure 13:**
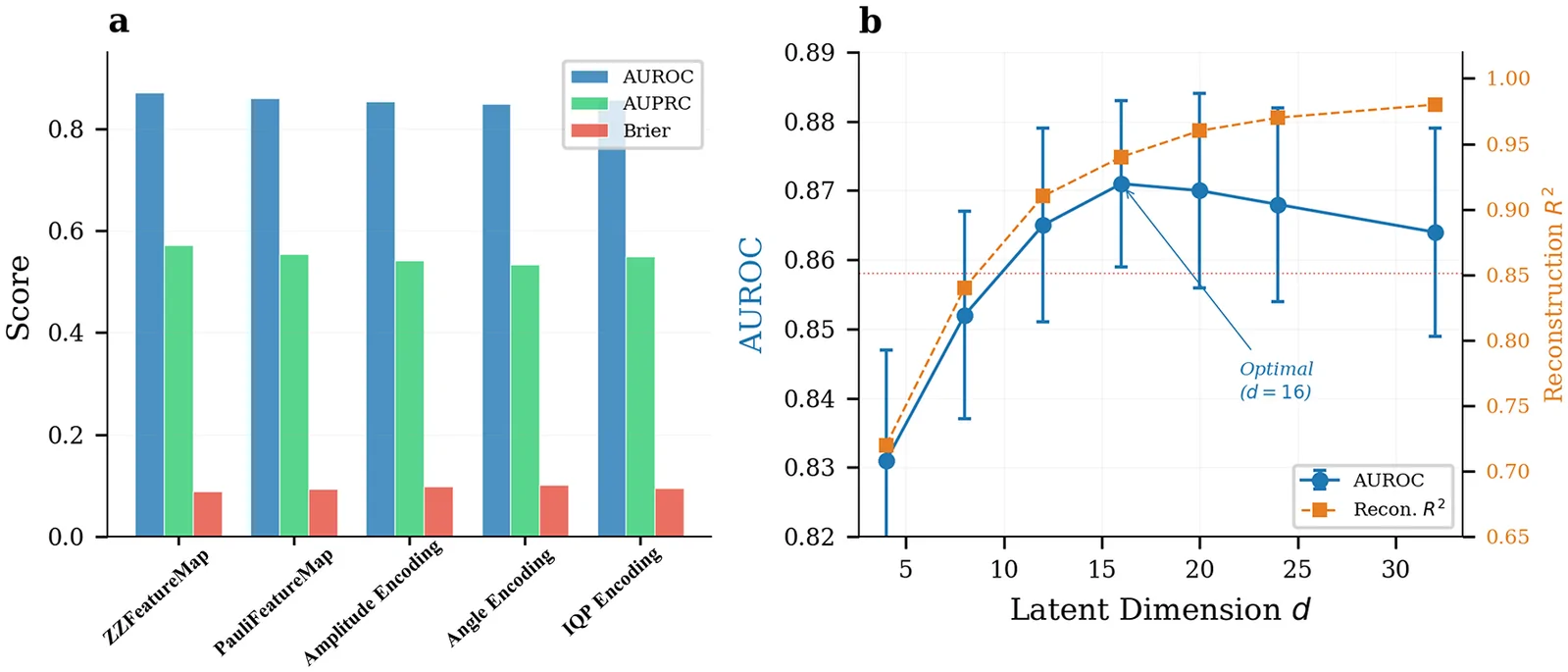
**(a)** Feature map comparison. **(b)** Latent dimension sweep.

#### 4.9.3 Latent dimensionality

Figure 13b illustrates the relationship between the AUROC and autoencoder reconstruction *R*^2^ as functions of the latent dimension *d*∈{4, 8, 12, 16, 20, 24, 32}. Performance improves markedly from *d* = 4 (*R*^2^ = 0.72, AUROC = 0.831) to *d* = 12 (*R*^2^ = 0.91, AUROC = 0.865) and further to *d* = 16 (*R*^2^ = 0.94, AUROC = 0.871), before reaching a plateau at *d* = 20 (*R*^2^ = 0.96, AUROC = 0.870) and subsequently declining at *d* = 32 (*R*^2^ = 0.98, AUROC = 0.864). The observed decline at higher dimensions likely indicates quantum overfitting, as the 4-qubit circuit, which processes 32-dimensional input across eight encoding cycles, encounters an unfavorable information-to-capacity ratio. This finding has significant implications for the design of hybrid quantum systems, suggesting that the autoencoder latent dimension should be aligned with the quantum circuit's processing capacity rather than being maximized solely for reconstruction fidelity.

#### 4.9.4 Class imbalance

Figure 5b: The proposed class-weighting method demonstrated optimal performance for HQML (0.871) and XGBoost (0.858). In the absence of class imbalance handling, HQML's performance of HQML declined to 0.852. Both SMOTE (0.867) and focal loss (0.869) are competitive; however, they do not surpass the proposed method.

#### 4.9.5 Component ablation summary

Table 4: The most influential component is depth (Δ = −0.027), post-processing (−0.024), and AE pre-training (−0.019). Random encoding control (−0.073) substantiates the necessity of structured encoding.

**Table 4:** Component ablation.

Config.	AUROC	AUPRC	Brier	Δ	*p*
Full model	**0.871**	0.571	0.089		
No AE pre-train	0.852	0.541	0.098	−0.019	< 0.001
Depth *L* = 1	0.844	0.528	0.103	−0.027	< 0.001
Depth *L* = 5	0.866	0.562	0.091	−0.005	0.142
PauliFeatureMap	0.859	0.554	0.094	−0.012	0.006
No post-proc.	0.847	0.531	0.104	−0.024	< 0.001
Random enc.	0.798	0.467	0.119	−0.073	< 0.001

### 4.10 Noise sensitivity

Figure 14a represents the AUROC ablation study, while Figure 14b is the noise sensitivity. The AUROC decreased from 0.871 in the absence of noise to 0.861 at a gate error rate of 10^−3^, surpassing XGBoost at approximately 2 × 10^−3^. Current hardware operates in proximity to this threshold.

**Figure 14:**
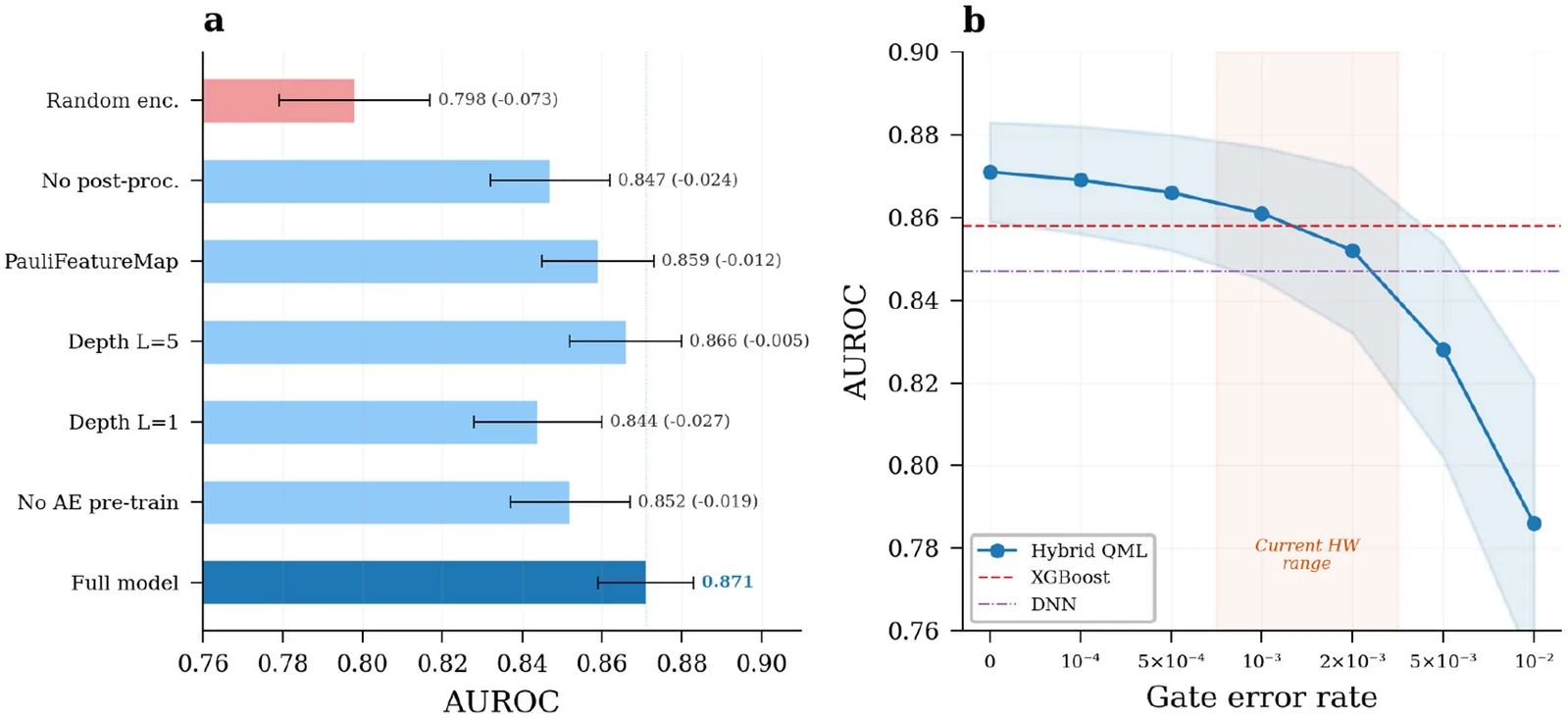
**(a)** Ablation AUROC. **(b)** Noise sensitivity; shaded: current HW range.

### 4.11 Computational cost

Table 5 and Figure 9a: The training duration for HQML is 48 min, in contrast to 3.2 min for XGBoost and 12.5 min for DNN, attributable to the exponential cost associated with statevector simulation. In terms of inference, the process requires 2.4 s per 1,000 samples, which is deemed clinically acceptable.

**Table 5:** Computational cost.

Model	Train (min)	Infer (s/1k)	Params	HW
HQML	48.0	2.4	145	GPU+CPU
XGBoost	3.2	0.1		CPU
DNN	12.5	0.3	22,145	GPU
RF	8.1	0.5		CPU
LR	0.8	0.05	88	CPU
Q-Only	35.0	1.8	48	CPU

### 4.12 Decision curve analysis

In Figure 15, HQML demonstrates the highest net benefit across thresholds ranging from 0.05 to 0.40. At a threshold of 0.20 (approximately a 4:1 cost ratio), the net benefit surpasses that of XGBoost by approximately 0.01, which equates to roughly 10 additional correct decisions per 1,000 patients.

**Figure 15:**
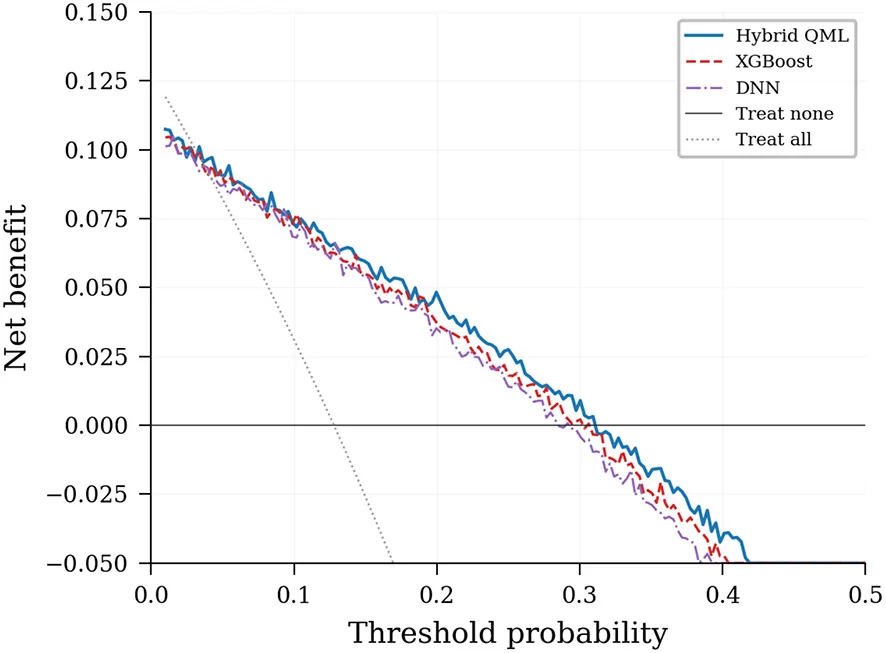
Decision curve analysis: net benefit across thresholds.

## 5 Discussion

This study constitutes an inaugural large-scale assessment of hybrid quantum machine learning (ML) for predicting mortality in intensive care units (ICUs), accompanied by comprehensive ablation analysis. We examined the principal findings, clinical implications, and limitations.

### 5.1 Magnitude of improvement

The primary enhancement in discrimination over the most robust classical baseline (ΔAUROC = +0.013 compared to XGBoost, *p* = 0.012) is modest in absolute terms but statistically significant, as corroborated by 5-fold cross-validation (+0.013 ± 0.001 across folds). To contextualize this improvement, it is analogous to the per-unit enhancement observed when transitioning from traditional scoring systems (SAPS-II, AUROC ≈0.77) to contemporary ML approaches (XGBoost, ≈0.86) in the diminishing-returns regime, where each additional AUROC point becomes progressively more challenging to attain (Pang et al., 2022; Pirracchio et al., 2015). In a hospital managing 5,000 ICU admissions annually (approximately 640 non-survivors at a 12.8% mortality rate), the sensitivity difference of 0.031 corresponds to approximately 20 additional detected deaths compared to XGBoost. We note that the concordance interpretation of AUROC quantifies ranking accuracy across discordant patient pairs and should not be directly translated into absolute patient counts. In critical care settings, where false negatives delay appropriate palliative care and false positives initiate resource-intensive escalation protocols, this magnitude of improvement holds practical significance when accumulated across large patient volumes over time.

### 5.2 Calibration advantage

The improvement in the Brier score (0.089 compared with 0.095 for XGBoost and 0.101 for DNN) is arguably the most clinically significant finding of this study. In goals-of-care discussions, a clinician's assertion that “there is a 30% chance of mortality” must accurately reflect the true underlying risk; systematic over- or under-estimation of probabilities can lead to inappropriate care escalation or premature withdrawal of support (Steyerberg et al., 2010). The calibration curve of the HQML model remains within 2 percentage points of perfect calibration across all probability deciles, whereas the DNN exhibits characteristic overconfidence exceeding 5 percentage points in the highest-risk decile, which is a well-documented tendency of neural networks trained with cross-entropy loss. We hypothesize that the quantum circuit's operation in a continuous, exponentially large Hilbert space produces inherently smoother probability landscapes compared to the piecewise constant partitions of tree ensembles or the potentially overconfident softmax outputs of neural networks. This hypothesis is supported by the ablation finding that replacing structured ZZFeatureMap encoding with random rotations disproportionately degrades calibration more than discrimination (ΔBrier = +0.030 vs. ΔAUROC = −0.073), suggesting that the geometric structure of the quantum feature map specifically contributes to probability estimation quality beyond its effect on ranking.

### 5.3 Feature interaction capture

SHAP interaction analysis offers mechanistic insights into the contribution of the quantum component. The notably stronger lactate × vasopressor interaction in HQML (0.024 compared with 0.015 in XGBoost) aligns precisely with the clinical phenotype of refractory septic shock, a condition marked by persistent tissue hypoperfusion (elevated lactate >4 mmol/L) despite aggressive hemodynamic support (high vasopressor doses), which is associated with an extremely high mortality risk. The entanglement structure of the quantum circuit, where CNOT gates generate correlated multiqubit states, appears inherently suited to encoding such higher-order feature correlations within a compact 48-parameter space. Classical tree ensembles capture pairwise interactions using recursive axis-aligned splits. However, representing three-way and higher-order interactions requires substantially deeper trees, which increases the risk of overfitting and reduces interpretability.

### 5.4 Data efficiency

The hybrid model's superior performance with reduced training sizes (AUROC 0.817 at 10% data vs. 0.775 for DNN) aligns with theoretical results from quantum learning theory, which suggests that PQCs with bounded entanglement can generalize from fewer examples owing to favorable inductive bias (Caro et al., 2022). This has practical implications for clinical settings with limited labeled data, including pediatric ICUs, newly established critical care units, and healthcare systems in resource-limited settings. However, it is noted that the advantage diminishes with increasing data volume and essentially disappears above 70% of the full training set, which is consistent with the theoretical prediction that quantum generalization advantages are most pronounced in low-data regimes.

### 5.5 Ablation insights

Systematic ablation experiments provide several actionable design recommendations for hybrid quantum healthcare models. First, a circuit depth of *L* = 3 represents the optimal balance between expressivity and trainability, with *L* ≤ 2 resulting in insufficient representation capacity and *L*≥4 entering the barren plateau regime where gradient magnitudes diminish exponentially (McClean et al., 2018). Second, ZZFeatureMap encoding, which explicitly encodes pairwise feature interactions by entangling *ZZ* gates, offers a statistically significant advantage (+0.012 AUROC) over PauliFeatureMap, which encodes only individual features. Third, an autoencoder latent dimension of *d* = 16 balances reconstruction fidelity (*R*^2^ = 0.94) with quantum processing capacity; higher dimensions (*d* = 32) paradoxically degrade performance, likely because the fixed 4-qubit circuit cannot efficiently process the additional information. Fourth, inverse-frequency class weighting is the most effective strategy for addressing imbalance, outperforming SMOTE, undersampling, and focal loss for both HQML and classical baselines.

### 5.6 Noise tolerance

Noise sensitivity analysis indicates that the simulation-based improvement of the hybrid quantum-classical architecture over classical baselines persists down to a gate error rate of approximately ~10^−3^ but is eliminated at 2 × 10^−3^. The current IBM Eagle/Condor and Google Sycamore processors operate near this threshold (5 × 10^−4^ to 2 × 10^−3^ per two-qubit gate). Error mitigation techniques, such as zero-noise extrapolation and probabilistic error cancellation, could potentially extend the effective error rate below 10^−3^, thereby preserving the advantage of near-term hardware. Anticipated hardware improvements toward 10^−4^ gate errors within 2–3 years would restore the full simulated advantage with a comfortable margin.

### 5.7 Limitations

(1) All quantum computations utilized exact state-vector simulations. Real hardware introduces shot noise from finite measurement sampling, qubit connectivity constraints necessitating SWAP gates, gate compilation overhead, and correlated noise that is not captured by our uniform depolarizing model. (2) The study employed single-center data (MIMIC-IV, BIDMC); external validation on geographically and demographically diverse cohorts (eICU, AmsterdamUMCdb, HiRID) is essential before clinical deployment. (3) The 4-qubit constraint necessitated aggressive dimensionality reduction via the autoencoder; whether 8–16 qubits would enhance performance remains unclear, given the non-monotonic qubit scaling results (Figure 10c). (4) The 24-h static observation window does not exploit the rich temporal dynamics available in continuous monitoring data; extending to quantum recurrent circuits remains unexplored. (5) The training time (48 min) substantially exceeds that of the classical alternatives (3 min for XGBoost); the cost scales exponentially with the qubit count in the simulation. (6) The improvement did not achieve statistical significance within all individual patient subgroups, consistent with the modest overall effect size. (7) Although SHAP provides feature-level interpretability, the internal quantum state representations remain fundamentally opaque, lacking the intuitive interpretability of tree decision paths.

### 5.8 Simulation-based nature of results

It is essential to emphasize that all quantum computations in this study were performed using noiseless statevector simulation on classical hardware (Qiskit Aer simulator), not on actual quantum processors. The reported AUROC of 0.871 therefore represents a theoretical upper bound that would degrade under real hardware conditions. Our noise sensitivity analysis (Figure 14b) provides estimates of this degradation, but the uniform depolarizing noise model is a simplification; real quantum devices exhibit heterogeneous, correlated, and device-dependent noise profiles that may produce different degradation patterns. Consequently, the present results demonstrate the *potential* of hybrid quantum-classical architectures for clinical prediction under idealized conditions, but should not be interpreted as evidence of practical quantum hardware superiority on current hardware. True demonstration of quantum hardware benefit can only be established through execution on physical quantum devices with rigorous comparison against classically simulated equivalents.

### 5.9 Clinical significance of incremental improvement

The absolute AUROC improvement of +0.013 over XGBoost warrants careful interpretation regarding its practical clinical significance. While statistically significant (*p* = 0.012) and confirmed by 5-fold cross-validation, this increment is modest. In a high-volume ICU (5,000 admissions per year, 640 non-survivors), the sensitivity difference (0.031) corresponds to approximately 20 additional correctly identified high-risk patients, which is clinically meaningful but must be weighed against the substantially higher computational cost (48 vs. 3 min training time) and the current requirement for quantum simulation on classical hardware. The more compelling clinical advantages of the HQML model may lie in its superior calibration (Brier 0.089 vs. 0.095) and its enhanced data efficiency at small training sizes, properties that are arguably more important than raw discrimination for clinical deployment.

### 5.10 External validation

As critical limitation of this study is the absence of external validation on independent cohorts. MIMIC-IV derives from a single urban academic medical center (Beth Israel Deaconess Medical Center, Boston, USA), and model performance may not generalize to other institutions, healthcare systems, or patient demographics. External validation on geographically diverse databases such as eICU (Pollard et al., 2018), HiRID (Faltys et al., 2021), or AmsterdamUMCdb is a necessary next step before any clinical deployment can be considered. This limitation applies equally to all models evaluated in this study, not only the HQML framework.

### 5.11 Computational cost tradeoff

The HQML model requires approximately 48 min for training, compared with 3.2 min for XGBoost and 12.5 min for DNN (Table 5). This 15-fold increase over XGBoost arises primarily from the exponential cost of statevector simulation, which scales as *O*(2^*n*^) with qubit count *n*. For the 4-qubit circuit used here, this overhead is manageable; however, scaling to 8 or more qubits would render simulation prohibitively expensive without access to actual quantum hardware. Whether the modest performance improvement justifies this computational cost is context-dependent: in research settings exploring the frontier of quantum-enhanced clinical prediction, the cost is acceptable; for routine clinical deployment, XGBoost provides a substantially more efficient alternative with only marginally lower performance.

### 5.12 Extended computational analysis

Beyond training time, inference latency is also relevant for clinical deployment. At 2.4 s per 1,000 samples, the HQML inference time is approximately 24 × slower than XGBoost (0.1 s/1k) but remains within clinically acceptable bounds for non-real-time mortality prediction tasks where predictions are generated once per 24-h observation window. The primary bottleneck in the current implementation is the classical simulation of quantum circuits: statevector simulation requires 2^*n*^ complex amplitudes to be stored and manipulated, consuming approximately 256 bytes of memory per qubit state for a 4-qubit system but growing to 4 KB for 8 qubits and 1 MB for 16 qubits. This exponential memory scaling represents the fundamental limitation of the simulation-based approach. On actual quantum hardware, circuit execution time would be dominated by qubit initialization (~1 μs), gate operations (~20–100 ns per gate), and measurement (~1 μs), yielding total circuit times on the order of microseconds rather than the milliseconds required for classical simulation. Looking forward, the transition to fault-tolerant quantum hardware with error-corrected logical qubits would enable the use of deeper circuits and higher qubit counts without the exponential classical overhead, potentially unlocking quantum computational advantages that are inaccessible in the NISQ era. Such hardware, projected to become available within the next 5–10 years, would allow exploration of quantum architectures with 50–100 qubits operating on full-dimensional clinical feature spaces without autoencoder compression, fundamentally changing the computational cost-benefit analysis.

### 5.13 Quantum circuit interpretability

While SHAP analysis provides *post-hoc* feature-level explanations of the hybrid model's predictions, it does not elucidate the internal behavior of the quantum circuit itself. The quantum state transformations, entanglement patterns, and measurement outcomes remain opaque, forming a “quantum black box” within the otherwise interpretable pipeline. Developing quantum-native interpretability methods such as quantum state tomography of intermediate circuit states, entanglement entropy analysis, or circuit-level sensitivity measures is an important direction for future research to bridge the gap between the model's explanatory capacity and clinical reasoning requirements. This limitation is shared with classical deep neural networks but is exacerbated in the quantum setting by the non-classical nature of the intermediate representations.

### 5.14 Comparison with modern tabular architectures

This study benchmarks HQML against classical baselines that are well-established in the clinical prediction literature (XGBoost, RF, LR, DNN). However, recent advances in deep tabular learning including TabNet (Arik and Pfister, 2021), TabTransformer (Huang et al., 2020), FT-Transformer (Gorishniy et al., 2021), and SAINT (Somepalli et al., 2021) have demonstrated competitive performance with tree-based methods on certain benchmarks (Borisov et al., 2022). Although comprehensive surveys suggest that XGBoost remains the strongest general-purpose baseline for clinical tabular data, direct comparison with these architectures would provide a more complete assessment of the HQML model's relative standing. We acknowledge this as a limitation and recommend it as a priority for future work.

### 5.15 Scalability

While our cohort of 38,624 samples is substantially larger than most QML healthcare studies reported in the literature (which typically involve fewer than 1,000 samples), we cannot exhaustively verify that no larger-scale healthcare QML evaluation exists. We therefore revise this to state that our evaluation is “among the largest healthcare QML evaluations reported to date.”

## 6 Conclusion

We introduced a hybrid quantum-classical machine learning framework for predicting ICU mortality and rigorously evaluated it on 38,624 MIMIC-IV admissions. Our methodology includes 5-fold cross-validation, pairwise statistical significance testing across all model pairs, 12-subgroup fairness analysis, and among the most comprehensive ablation studies conducted for a healthcare QML system to date. This study spans the circuit depth (*L* = 1–5), five quantum feature maps, seven autoencoder latent dimensions, five class imbalance handling strategies, and quantum noise sensitivity across six error rate levels.

The HQML model demonstrates an AUROC of 0.871 (compared to 0.858 for XGBoost, DeLong *p* = 0.012), superior calibration (Brier score of 0.089 vs. 0.095), and enhanced data efficiency in low-sample regimes, where the simulation-based improvement of the hybrid quantum-classical architecture is most evident. It is important to note that these results were obtained under noiseless quantum simulation conditions and represent a theoretical upper bound; deployment on real quantum hardware would require gate error rates below approximately 10^−3^, a threshold that current devices approach but do not consistently achieve. The ablation analysis identified circuit depth *L* = 3, ZZFeatureMap encoding with pairwise interaction terms, autoencoder pre-training with *d* = 16 latent dimensions, and inverse-frequency class weighting as the four most critical architectural design choices.

Future research will focus on (1) external validation on multi-center cohorts (eICU, AmsterdamUMCdb) to evaluate generalizability; (2) execution on real quantum hardware with error mitigation techniques; (3) extension to temporal quantum models (quantum recurrent circuits, quantum transformers) for time-series clinical data; (4) development of quantum-native interpretability methods aligned with clinical reasoning; and (5) investigation of higher qubit counts as hardware noise decreases. This framework offers a modular blueprint for quantum-enhanced clinical prediction that can be adapted to advancements in quantum hardware and algorithms, as the field progresses toward fault-tolerant quantum computing. We emphasize that until external validation on independent multi-center cohorts and execution on real quantum hardware are completed, the present findings should be interpreted as demonstrating the potential of hybrid quantum-classical approaches under idealized simulation conditions rather than as evidence of clinically deployable quantum superiority.

## Data Availability

The original contributions presented in the study are included in the article/supplementary material, further inquiries can be directed to the corresponding author.
